# Case report: A novel heterozygous synonymous variant in deep exon region of *NIPBL* gene generating a non-canonical splice donor in a patient with cornelia de lange syndrome

**DOI:** 10.3389/fgene.2022.1056127

**Published:** 2022-11-23

**Authors:** Meizhen Shi, Yuying Liang, Bobo Xie, Xianda Wei, Haiyang Zheng, Chunrong Gui, Rong Huang, Xin Fan, Chuan Li, Xiaojiao Wei, Yunting Ma, Shaoke Chen, Yujun Chen, Baoheng Gui

**Affiliations:** ^1^ Center for Medical Genetics and Genomics, The Second Affiliated Hospital of Guangxi Medical University, Nanning, China; ^2^ The Guangxi Health Commission Key Laboratory of Medical Genetics and Genomics, The Second Affiliated Hospital of Guangxi Medical University, Nanning, China; ^3^ Department of Pediatrics, The Traditional Chinese Medicine Hospital of YuLin, Yulin, China; ^4^ Department of Pediatrics, The Second Affiliated Hospital of Guangxi Medical University, Nanning, China

**Keywords:** NIPBL, synonymous variant, non-canonical splice donor, whole-exome sequencing, cornelia de lange syndrome

## Abstract

Cornelia de Lange syndrome (CdLS) is an autosomal dominant or X-linked genetic disease with significant genetic heterogeneity. Variants of the *NIPBL* gene are responsible for CdLS in 60% of patients. Herein, we report the case of a patient with CdLS showing distinctive facial features, microcephaly, developmental delay, and growth retardation. Whole exome sequencing was performed for the patient, and a novel *de novo* heterozygous synonymous variant was identified in the deep region of exon 40 in the *NIPBL* gene (NM_133433.4: c. 6819G > T, p. Gly2273 = ). The clinical significance of the variant was uncertain according to the ACMG/AMP guidelines; however, based on *in silico* analysis, it was predicted to alter mRNA splicing. To validate the prediction, a reverse transcriptase-polymerase chain reaction was conducted. The variant activated a cryptic splice donor, generating a short transcript of *NIPBL.* A loss of 137 bp at the 3′ end of *NIPBL* exon 40 was detected, which potentially altered the open reading frame by inserting multiple premature termination codons. Quantitative real-time PCR analysis showed that the ratio of the transcription level of the full-length transcript to that of the altered short transcript in the patient was 5:1, instead of 1:1. These findings may explain the relatively mild phenotype of the patient, regardless of the loss of function of the truncated protein due to a frameshift in the mRNA. To the best of our knowledge, this study is the first to report a synonymous variant in the deep exon regions of the *NIPBL* gene responsible for CdLS. The identified variant expands the mutational spectrum of the *NIPBL* gene. Furthermore, synonymous variations may be pathogenic, which should not be ignored in the clinical and genetic diagnosis of the disease.

## Introduction

Cornelia de Lange syndrome (CdLS; OMIM #122470, 300590, 610759, 300882, and 614701) is a genetically heterogeneous autosomal dominant or X-linked dominant congenital multisystem disorder (Liu and Krantz, 2009; Boyle et al., 2015). Typical clinical manifestations of CdLS include distinctive craniofacial features (long philtrum, micrognathia, low-set ears, synophrys, myopia, long curly eyelashes, ptosis, anteverted nostrils, thin upper lip, high arched palate, widely spaced teeth, and short neck), growth retardation, behavioral abnormalities, and upper extremity defects (Boyle et al., 2015). International guidelines with scoring criteria have been published for the clinical diagnosis of CdLS (Kline et al., 2018). Hence, classic CdLS is easily recognized by experienced pediatricians and clinical geneticists because of the unique craniofacial appearance and growth pattern, as well as limb deformities observed in these patients. However, accumulating evidence shows a remarkable phenotypic heterogeneity among CdLS patients (Yuan et al., 2015; Kline et al., 2018). Hence, the genetic method remains an important diagnostic modality of CdLS. The prevalence of CdLS is estimated to be between 1:10 000 and 1:50 000 in live births (Mannini et al., 2013). However, the actual number of cases is expected to be higher, because some patients with atypical symptoms may not have been clinically diagnosed.

The cohesins are important regulators, which mainly maintain genomic stability, separate chromosomes and chromatin structure, and regulate gene expression (Kamada and Barilla, 2018; Gao et al., 2019). CdLS is mainly attributable to the pathogenic variations of the genes encoding cohesin complexes, altering the levels and patterns of gene expression during development (Liu et al., 2020). The core components of cohesin RAD21, SMC1A and SMC3 proteins are considered to form a tripartite ring wrapped chromatids. The NIPBL protein is essential to mediate the loading of cohesin onto chromosomes (Muto et al., 2014). The chromatin associated protein BRD4 can enhance the load of NIPBL protein by binding with acetylated histone H3 Lys27 and targeting the enhancer clusters (Hnisz et al., 2013). The HDAC8 protein regulates the release of cohesin complexes from chromatin by deacetylating SMC3 protein (Kline et al., 2018). At present, six causing genes of CdLS have been reported, and approximately 60% of CdLS patients have pathogenic variants in the *NIPBL* (OMIM #608667) gene (Kline et al., 2018), The *NIPBL* gene contains 47 exons located at 5p13.2, encoding two delangin subtypes A and B (Nipped-B-like protein) with 2804 and 2697 amino acids (Krantz et al., 2004). About 10% of cases are caused by variants in the five other genes belonging to the cohesin pathway, including *SMC1A*, *SMC3*, *HDAC8*, *RAD21*, and *BRD4* (Boyle et al., 2015; Olley et al., 2018). However, the underlying genetic causes for the remaining 30% of cases are still unknown (Mannini et al., 2013; Boyle et al., 2015; Watrin et al., 2016).

To date, the professional Human Gene Mutation Database (HGMD) has reported more than 500 diverse variations in the *NIPBL* gene in CdLS patients, most of which are missense, nonsense, frameshift, or classical splicing variants. The genotype-phenotype correlations between these *NIPBL* variants and CdLS are relatively clear. However, little is known about these rare disease-causing variations, especially synonymous variants. To date, only one synonymous *NIPBL* variant has been reported in a fetal case (Qiao et al., 2021). Few recognizable features were observed during the prenatal stage in the patient carrying a synonymous variant, and only skeletal dysplasia of the bilateral upper extremity and congenital heart defects were observed in the fetus (Qiao et al., 2021). Thus, synonymous variants in *NIPBL* and their genotype-phenotype correlation with CdLS remain unelucidated.

Here, we report a case of CdLS with distinctive facial features, microcephaly, developmental delay, and growth retardation. A novel *de novo* heterozygous synonymous variant c.6819G>T p (Gly2273 = ) deep in the exon 40 of the *NIPBL* gene was identified by whole exome sequencing (WES). Further validation studies showed that this synonymous variant altered the splicing mode of *NIPBL*, thereby producing an abnormally shorter transcript. Consequently, our study elucidated the genetic etiology of the patient and provided a theoretical basis and guidance for reproductive genetic counseling and prenatal diagnosis for the family. Furthermore, the mutational spectrum of the *NIPBL* gene was enriched, deepening our understanding of the disease and its diverse genetic causes.

## Materials and methods

### Ethics statement

This study was approved by the Ethics Committee of the Second Affiliated Hospital of Guangxi Medical University (No. 2019-106) and complied with the principles of the Declaration of Helsinki. Samples and information were collected after obtaining written informed consent from parents.

### Case report

**FIGURE 1:**
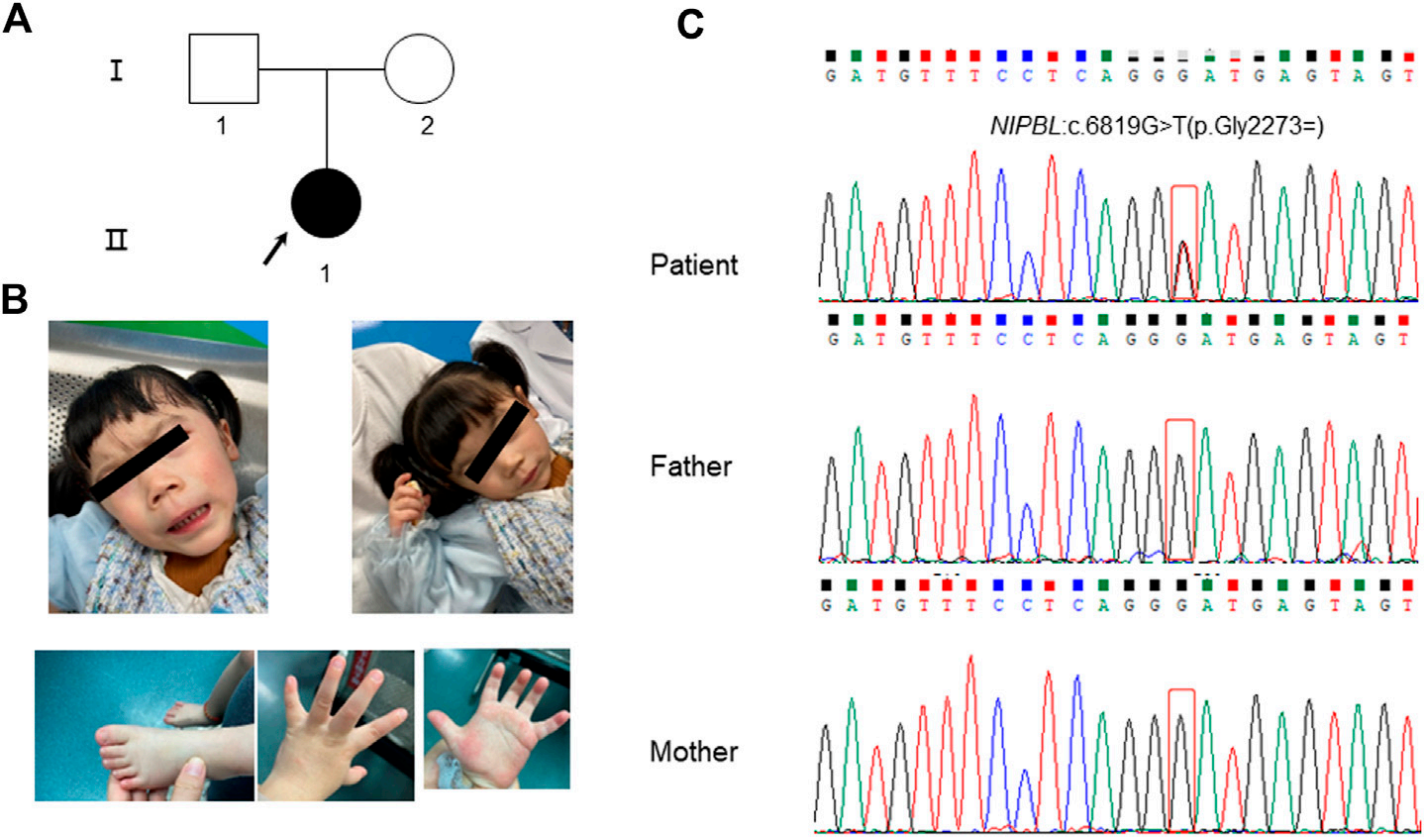
Clinical features of the patient and Sanger sequencing results of her family members. **(A)** Pedigree of the family. The arrow indicates the patient. **(B)** The patient presented with typical facial features of Cornelia de Lange syndrome (CdLS). **(C)** The results of Sanger sequencing for the patient and her parents. A heterozygous synonymous variant of *NIPBL* (NM_133433.4): c.6819G>T (p.Gly2273 = ) was detected in the patient while her parents showed the wild-type variant.

The patient was a full-term female infant who showed patent foramen ovale after birth with a weight of 3.97 kg and length of 43 cm, no history of neonatal asphyxia, and no difficulties during breastfeeding. The patient’s parents were healthy and nonconsanguineous (Figure 1A). Prenatal ultrasound examination conducted at week 24 of gestation suggested intrauterine growth retardation (approximately 2–4 weeks of delay). She raised her head at 3 months, sat without help at 7 months, and walked without aid at 1 year and 8 months, indicating developmental retardation. When the patient was 3 years and 7 months old, she showed an apparent short stature with height of 85.5 cm (−3.8SD) and weight of 8 kg. Other clinical symptoms included microcephaly (head circumference: 43 cm), language and intelligence retardation, short fifth finger, and dysmorphic facial features (inverted triangular face, long black eyelashes, synophrys, low nose bridge, wide eye distance, micrognathia, and long philtrum) (Figure 1B; Table 1).

**TABLE 1:** Diagnostic algorithm as suggested by the Consensus Statement.

Clinical features of CdLS	Score	Qiao et al. (2021)	This study
Synophrys (HP:0000664) and/or thick eyebrows (HP:0000574)	2	-	2
Short nose (HP:0003196), concave nasal ridge (HP:0011120) and/or upturned nasal tip (HP:0000463)	2	-	-
Long (HP:0000343) and/or smooth philtrum (HP:0000319)	2	-	2
Thin upper lip vermilion (HP:0000219) and/or downturned corners of mouth (HP:0002714)	2	-	-
Hand oligodactyly (HP:0001180) and/or adactyly (HP:0009776)	2	-	-
Congenital diaphragmatic hernia (HP:0000776)	2	-	-
Suggestive Features		-	
Global developmental delay (HP:0001263) and/or intellectual disability (HP:0001249)	1	-	1
Prenatal growth retardation (<2 SD) (HP:0001511)	1	1	-
Postnatal growth retardation (<2 SD) (HP:0008897)	1	-	1
Microcephaly (prenatally and/or postnatally) (HP:0000252)	1	-	1
Small hands (HP:0200055) and/or feet (HP:0001773)	1	-	-
Short fifth finger (HP:0009237)	1	-	1
Hirsutism (HP:0001007)	1	-	-
Total score		1	8

Classic CdLS: Total Score ≥11 points, of which at least 3 are cardinal features; Non-classic CdLS: Total Score between 9 or 10 points, of which at least 2 are cardinal features; molecular testing for CdLS, indicated; Total Score between four to eight points, of which at least 1 is cardinal feature; insufficient to indicate molecular testing for CdLS: Score <4 points.

### Whole exome sequencing and validation

Genomic DNA (gDNA) was extracted from peripheral blood leukocytes of the patient and her parents according to standard methods (QIAGEN, Germany). WES was performed with patient samples, followed by data filtration and analysis as previously described (Yu et al., 2021). The pathogenicity of the variants was analyzed according to ACMG/AMP variant interpretation standards and guidelines (Richards et al., 2015). SpliceAI (Jaganathan et al., 2019) (https://github. com/Illumina/SpliceAI), an *in silico* tool, was used to predict the effect of a candidate variant on splicing. The identified heterozygous synonymous variant of the *NIPBL* gene was validated in the patient and her parents by Sanger bidirectional sequencing using the forward primer 5′-cca​ttg​agc​cag​aac​act​agc-3′ and reverse primer 5′- ttg​cag​taa​tca​taa​ccc​aag​aga-3′. The *NIPBL* genomic reference sequence was downloaded from the UCSC Genome Browser (http://genome.ucsc.edu/, GRCh37/hg19, NM_133433.4).

### RNA transcript analysis by reverse transcriptase-polymerase chain reaction (RT-PCR) and quantitative real-time PCR (qPCR)

Total RNA was extracted from the leukocytes of the patient and her parents using the standard TRIzol method (Rio et al., 2010). cDNA was synthesized from RNA using the RevertAid First Strand cDNA Synthesis Kit (Thermo Fisher Scientific, United States). Two pairs of specific primers (A and B) targeting exon 40 and nearby exons of the *NIPBL* gene were designed to amplify the cDNA library (forward primer A:5′-tgc​ctt​tat​tca​gca​tcc​aag​t-3′ and reverse primer A:5′-ctt​gtt​ccg​cat​agc​agg​ttc​t-3′; forward primer B:5′-ttgcctttattcagcatccaag-3′ and reverse primer B:5′-ggactcgtcttgtctgaaaccc-3′) to obtain the target PCR fragments. The RT-PCR products were separated by 1.5% agarose gel electrophoresis to detect potentially altered transcripts and were further analyzed by Sanger sequencing *via* band separation on an agarose gel to determine the exact sequence. To investigate the levels of full-length normal transcripts and altered short transcripts in the patient, different specific primers targeting these two transcripts were designed for qPCR analysis. Primer WT could only amplify normal transcripts (forward primer: 5′-cca​tca​tgc​agc​ttt​atc​tca​a-3′ and reverse primer: 5′-ctt​gtt​ccg​cat​agc​agg​ttc​t-3′), and primer MUT could only amplify altered short transcripts (forward primer: 5′-aaa​acc​tcc​aga​cct​acc​tac​aag​a-3′ and reverse primer: 5′-ata​tgg​cac​aca​ctg​agg​aaa​ca-3′). *GAPDH* was used as the internal reference gene (forward primer: 5′-agc​cac​atc​gct​cag​aca​c-3′ and reverse primer: 5′-gcc​caa​tac​gac​caa​atc​c-3′).

## Results

The patient showed synophrys and thick eyebrows, long and smooth philtrum, global developmental delay, intellectual disability, microcephaly, short fifth finger, and postnatal growth retardation (<2 SD) (Figure 1B; Table 1). The patient was clinically diagnosed with CdLS (total clinical score: eight points, Table 1) following the guidelines of international scoring criteria for CdLS (Kline et al., 2018). However, specific molecular tests are required.

During the filtering and analysis of candidate disease-causing variants performed using WES, including all those identified in known CdLS genes, no pathogenic or likely pathogenic variants that could explain the phenotype of the patient were identified. However, we noticed a heterozygous synonymous variation located in a deep region of exon 40 in the *NIPBL* gene (chr5:37049268; c.6819G>T; p. Gly2273 = ), which has not been previously reported. Validation using Sanger sequencing indicated that the variation occurred *de novo* in the patient (Figure 1C). In addition, the variation was not found in the 1000 Genomes, ExAC, genomAD, and dbSNP databases. And we found a synonymous variant at the same genomic position, but with a different nucleotide change (G>A) is reported on gnomAD (allele frequency 0.000003979) (https://gnomad.broadinstitute.org/variant/5-37049268-G-A?dataset=gnomad_r2_1). According to the ACMG/AMP variant interpretation standards and guidelines (Richards et al., 2015), this variant was characterized as that of uncertain significance based on the above evidence (PM2 and PM6).

**FIGURE 2:**
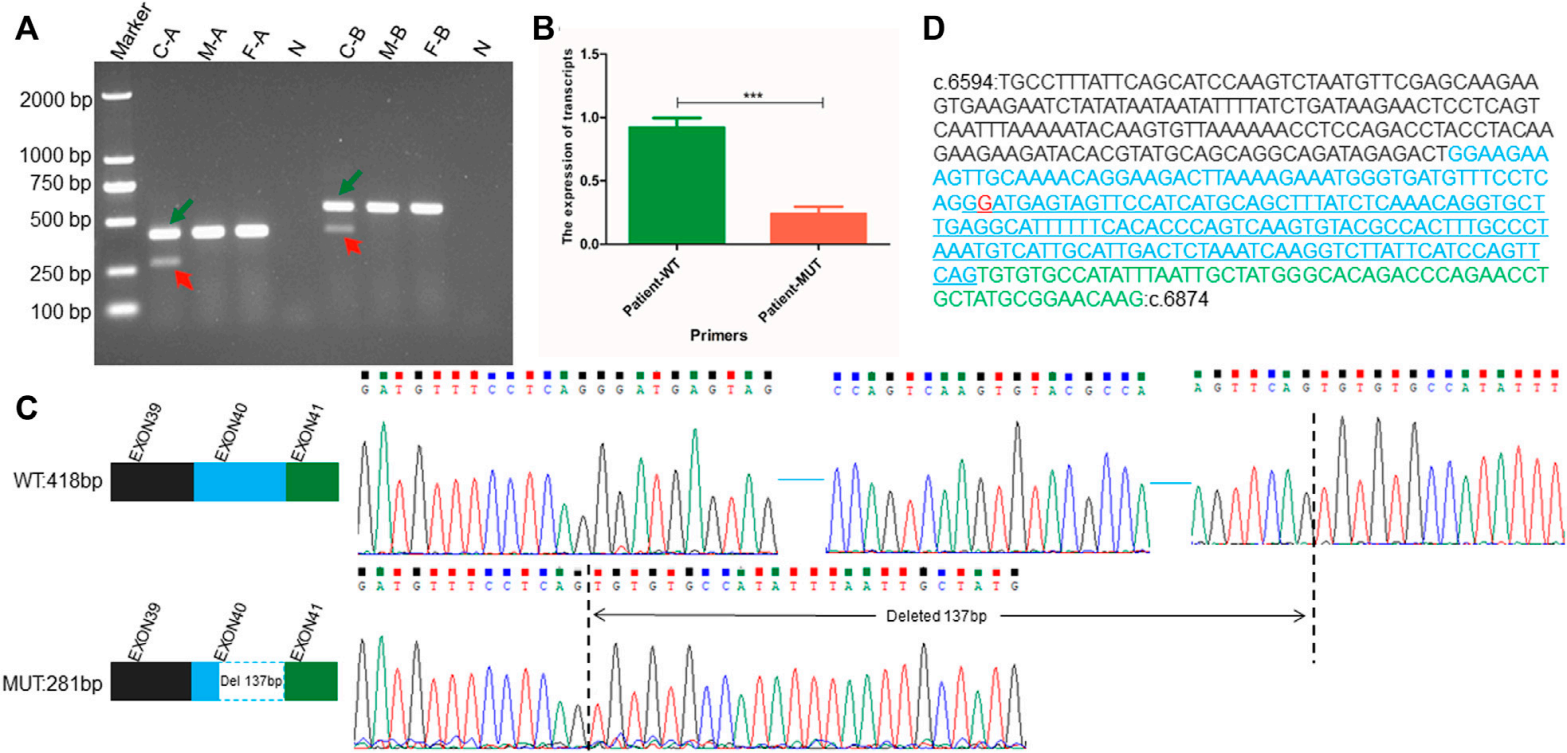
RNA transcript analysis performed using RT-PCR, qPCR, and Sanger sequencing. **(A)** Agarose gel electrophoresis showing RT-PCR results using primer pairs A and B targeting the exonic regions flanking the position of the *NIBPL de novo* synonymous variant (c.6819G>T (p.Gly2273 = ) in exon 40. Green arrows point to normal transcripts (wild-type transcript), and red arrows point to altered short transcripts (variant transcript). “C” represents the patient, “F” represents the father of the patient, “M” represents the mother of the patient, “N” represents the negative control derived using the gDNA of the patient as an amplification template, “A” represents the pair A, and “B” represents the pair B amplification primer **(B)** The transcription level of wild-type full-length transcript to altered mutant short transcript in *NIPBL*. WT represents the unique sequence of wild-type transcript, which can only specifically amplify the wild-type transcript; MUT represents the unique sequence of the altered short mutant transcript, which can only specifically amplify the abnormal transcript attributed to the c.6819G>T variant. **(C)** Patterns and sequences of wild-type transcript and altered short transcript attributed to variation. The upper left represents the splicing pattern of wild-type cDNA, and the upper right represents the exact sequence of wild-type cDNA splicing. The blue horizontal line represents the omitted wild-type sequence. The lower left represents the altered splicing pattern of cDNA attributed to variation (The blue dashed box indicates 137 bp deletion at the 3′ end of exon 40 in *NIPBL*), and the lower right represents the exact sequence obtained after altered splicing of cDNA attributed to variation. The vertical black dotted line indicates the sequence position of abnormal splicing deletion **(D)** The exact sequence of cDNA amplified by primer Pair A. The blue underlined sequence indicates the deletion of a 137 bp sequence. The black font represents the partial base sequence of exon 40, the blue font represents the base sequence of exon 40, and the green font represents the partial base sequence of exon 41. The red base G represents the mutation site.

The c.6819G>T synonymous variant was further predicted to influence the *NIPBL* gene splicing by activating a new donor site, according to SpliceAI analysis. Subsequently, RT-PCR analysis confirmed the presence of two types of *NIPBL* transcripts in the patient: one was the normal longer transcript (418 bp) and the other was the altered shorter transcript (281 bp). In contrast, only the normal longer transcript (418 bp) was present in the parents (Figure 2A). To investigate the exact sequences of the different transcripts, RT-PCR products of the patient and her parents were analyzed by Sanger sequencing. As expected, the shorter transcript (281 bp) in the patient was derived from the mutated allele, whereas the longer sequence (418 bp) in the patient and her parents represented the wild-type transcript. Sequence analysis confirmed that the c.6819G>T variation in the deep region of exon 40 generated a non-canonical splice donor, resulting in a 137 bp deletion at the 3’ end of exon 40 (Figures 2C,D). The partial loss of exon 40 presumably altered the downstream open reading frame of *NIPBL*, further generating multiple preterm termination codons. In addition, according to the ExAC database, the probability of loss-of-function intolerance (pLI) is 1, indicating that *NIPBL* is highly intolerant to loss-of-function variations of heterozygosity. These findings provide additional crucial evidence (PVS1) to support the interpretation of the identified variant as pathogenic according to the ACMG/AMP variant interpretation standards and guidelines. This variant was submitted to ClinVar (ID: SCV002586369).

The patient is heterozygous for a synonymous c.6819G>T variant. Unexpectedly, as shown by agarose gel electrophoresis, the ratio of the transcription levels of the wild-type longer transcript to the altered shorter transcript was not exactly 1:1 (Figure 2A). Further qPCR analysis targeting these two transcripts showed that the transcription ratio of normal full-length transcripts to altered shorter transcripts was approximately 5:1 (Figure 2B).

## Discussion

Cornelia de Lange syndrome (CdLS) is a rare congenital developmental disorder with multi-organ system involvement and genetic heterogeneity. The primary symptoms include facial dysmorphisms, such as long and thick eyelashes, synophrys and hypertrichosis of the brows, thin lips with downturned corners, a depressed nasal bridge with anteverted nares, widely spaced teeth and micrognathia, hirsutism, cutis marmorata, growth retardation, retarded psychomotor development, hand oligodactyly and/or hand foot, and other major deformities (Kline et al., 2018; Li et al., 2020). Herein, we report a patient with microcephaly, long black eyelashes, synophrys, low nose bridge, wide eye distance, micrognathia, long philtrum, growth retardation, and intellectual impairment. The clinical symptoms of the patient (clinical score: 8 points) partially overlapped with those of patients with typical CdLS (clinical score: ≥11 points) (Table 1) (Kline et al., 2018). Genetic analysis of genes related to CdLS, such as *NIPBL,* is essential for disease diagnosis. Truncating variants of *NIPBL* primarily lead to typical CdLS with more severe clinical symptoms, such as typical facial features, severe developmental and cognitive retardation, severe growth retardation, and structural abnormalities of the limb and other organs (Yan et al., 2006; Ansari et al., 2014). Missense mutations are mainly associated with milder phenotypes, characterized by the absence of limb abnormalities and involvement of mild developmental and growth retardation (Bhuiyan et al., 2006; Selicorni et al., 2007).

Few studies have focused on the pathogenic mechanisms of these unusual variations, particularly synonymous variants. This is mainly because synonymous variants are often considered benign and filtered out in routine clinical analysis, or they cannot be characterized as pathogenic or by a pathogenic grade according to the ACMG/AMP guidelines (Richards et al., 2015), which is of little significance for clinical diagnosis. However, accumulating evidence has shown that some specific synonymous variations can lead to diseases by influencing the mRNA stability and alternative splicing of exons (Nackley et al., 2006; Sauna and Kimchi-Sarfaty, 2011), which should garner more interest. To date, only one fetal case with a *NIPBL* synonymous variant has been reported, providing limited phenotypic information related to a specific variant (Qiao et al., 2021). This is presumably a result of the difficulty in recognizing fetal features *in utero* or the late onset of clinical manifestations, which do not appear during the early developmental stage. The case we report here provides more comprehensive phenotypic information for CdLS patients (Table 1) with synonymous variants of the *NIPBL* gene, facilitating further studies on the relationship between synonymous variants and clinical phenotypes.

Alternative splicing mediated by synonymous variations is primarily observed several bases upstream or downstream of the exon-intron junction. These boundary sequences of exon-introns are the main recognition sites of the spliceosome (Krawczak et al., 2007), providing canonical splicing donors and receptors, which are typically highly conserved (Anna and Monika, 2018). Most variants in these typical loci can change the splicing of exons and introns, resulting in specific diseases. Hence, to a large extent, they are unlikely to be ignored in routine analysis, as in the previously reported CdLS case with a synonymous variant at the last base of exon 27 in the *NIPBL* gene (Qiao et al., 2021). Diseases caused by synonymous variations in deep exonic or introns are uncommon. The patient with CdLS reported here carries a novel heterozygous synonymous variant of *NIPBL* (chr5:37049268; c.6819G>T; p. Gly2273 = ) located in the middle region of exon 40. The results of RT-PCR analysis implied that the variation activated a cryptic donor splice site in *NIPBL*, resulting in a 137 bp deletion at the 3’ end of exon 40 (Figures 2C,D), which is consistent with the findings of a previous study (Olinger et al., 2021). This deletion may alter the downstream open reading frame of *NIPBL*, further generating multiple preterm termination codons. Interestingly, agarose gel electrophoresis indicated different transcription levels of the wild-type and altered short transcripts (Figure 2A). This was further confirmed by qPCR analysis, which showed an approximate 5:1 ratio for the two transcripts (Figure 2B). The decreased levels of the mutant transcript may be attributed to nonsense-mutation mediated decay. Decreased expression or activity of the NIPBL protein largely contributes to CdLS (Tonkin et al., 2004; Hulinsky et al., 2005; Zuin et al., 2017), suggesting that haploinsufficiency of *NIPBL* may be the pathogenic mechanism underlying CdLS. However, more functional studies are required to further explore the specific mechanism.

The patient in this study showed relatively milder symptoms than typical CdLS features, which may be explained by the relatively lower transcription level of the altered short transcript (less than 20%) (Figure 2B). This is consistent with previous findings showing that patients with more severe phenotypes have low levels of wild-type *NIPBL*, while those with milder symptoms have high levels of wild-type *NIPBL* (Kaur et al., 2016).

The accurate molecular genetic diagnosis of patients with CdLS is of great significance. However, 30% of patients with CdLS are not clearly diagnosed (Mannini et al., 2013; Boyle et al., 2015). Currently, the clinical practice of sequence variation interpretation mainly focuses on missense, nonsense, or typical splice variants (Richards et al., 2015). However, synonymous variations are usually considered “silent” in most cases because they do not alter the translated protein sequence (Gaither et al., 2021) to some extent, contributing to a missed diagnosis in some conditions. This may explain the negative findings in the WES analysis for the patient we reported when she was referred to other clinics, where the synonymous variation in *NIPBL* was likely to be ignored. To the best of our knowledge, this is the first report of a CdLS case with a synonymous variant in the deep exon region of the *NIPBL* gene. Furthermore, the identified variant enriched the mutational spectrum of *NIPBL* and deepened our understanding of the roles of synonymous variations in CdLS. The effects of synonymous variations on diseases should be examined in detail, and more caution is required when annotating these variants in routine genetic analysis.

## Data Availability

The original contributions presented in the study are publicly available. This data can be found here: https://db.cngb.org/search/project/CNP0003582/.
